# Increased copy-number variant load of associated risk genes in sporadic cases of amyotrophic lateral sclerosis

**DOI:** 10.1007/s00018-024-05335-8

**Published:** 2024-07-27

**Authors:** Maria Guarnaccia, Giovanna Morello, Valentina La Cognata, Vincenzo La Bella, Francesca Luisa Conforti, Sebastiano Cavallaro

**Affiliations:** 1 Institute for Biomedical Research and Innovation, National Research Council, P. Gaifami 18, Catania, 95126 Italy; 2 Department of Experimental Biomedicine and Advanced Diagnostics, ALS Clinical Research Center, Laboratory of Neurochemistry, University of Palermo, Palermo, Italy; 3 Department of Pharmacy, Health and Nutritional Sciences, University of Calabria, 87036 Rende, Italy

**Keywords:** Amyotrophic lateral sclerosis, Copy number variant, Customized aCGH, Diagnostics, CNVs load

## Abstract

Amyotrophic lateral sclerosis (ALS) is an age-related neurodegenerative disease characterized by selective loss of motor neurons in the brainstem and spinal cord. Several genetic factors have been associated to ALS, ranging from causal genes and potential risk factors to disease modifiers. The search for pathogenic variants in these genes has mostly focused on single nucleotide variants (SNVs) while relatively understudied and not fully elucidated is the contribution of structural variants, such as copy number variations (CNVs). Here, we applied an exon-centric aCGH method to investigate, in sporadic ALS patients, the load of CNVs in 131 genes previously associated to ALS. Our approach revealed that CNV load, defined as the total number of CNVs or their size, was significantly higher in ALS cases than controls. About 87% of patients harbored multiple CNVs in ALS-related genes, and 75% structural variants compromised genes directly implicated in ALS pathogenesis (*C9orf72*,* CHCHD10*,* EPHA4*,* FUS*,* HNRNPA1*,* KIF5A*,* NEK1*,* OPTN*,* PFN1*,* SOD1*,* TARDBP*,* TBK1*,* UBQLN2*,* UNC13A*,* VAPB*,* VCP*). CNV load was also associated to higher onset age and disease progression rate. Although the contribution of individual CNVs in ALS is still unknown, their extensive load in disease-related genes may have relevant implications for the diagnostic, prognostic and therapeutical management of this devastating disorder.

**Supplementary Information:**

The online version contains supplementary material available at 10.1007/s00018-024-05335-8.

## Introduction

Amyotrophic lateral sclerosis (ALS) (Mondo:0004976: Omim: PS105400) is a fatal neurodegenerative disorder with phenotypic and genetic heterogeneity [1, 2]. In addition to progressive voluntary muscle weakness, due to the loss of motor neurons in brain and spinal cord, ALS may involve cognitive and behavioral changes and frontotemporal dementia [2, 3]. Approximately 90% of ALS cases occur randomly as sporadic (sALS), while the remaining 10% have a family history of disease (fALS), with no clear clinical or pathological distinction [4]. Although the pathophysiology of ALS is unclear, about 85% of cases may be explained by a genetic cause [5]. While approximately 70% of the genetic mutations accounting for fALS have been identified, no significant genetic variations are associated to the majority of sALS (85%), highlighting a complex genetic heterogeneity underlying the sporadic cases. Although this may indicate a minor genetic contribution to sALS, estimates of high heritability support the search for additional genetic contributors in people with no apparent family history [6].

Since the discovery of the first familial ALS gene *SOD1* [7], over a hundred different genes have been associated with ALS, ranging from causative genes to potential risk factors and disease modifiers [1, 5, 8]. The search for pathogenic variants in these genes has primarily focused on single nucleotide variants (SNVs), while few studies have investigated the presence of both gross and micro structural variants, such as copy number variations (CNVs). These latter, ranging from 50 bp to several Mb [9], represent an important source of human genome variability [10] and might account for some of the missing heritability in sALS [8, 10, 11]. Most of the previous attempts to identify CNVs in ALS utilized high-density genome-wide single nucleotide polymorphism (SNP) arrays and were restricted to a set of tagSNP markers (about 317,000) derived from the Phase I of the International HapMap Project [12–14]. These platforms, targeting mainly common genomic polymorphisms with a median spacing of 5.5 kb, do not represent the most adequate strategies to fully characterize CNVs in human genome with a high resolution. Indeed, the majority of tagSNPs lie within noncoding regions, imposing a challenge to study their role in disease pathology. Moreover, regarding ALS-related genes, tagSNP markers cover very few exonic or intronic sequences with a median spacing of 15.45 kb (Supplementary materials S3_Table 3) and therefore are not sufficient to investigate both gross and small-scale CNVs in these regions.

Here, we designed a method to identify and characterize the complete repertoire of CNVs in ALS-related genes [15–17]. In particular, we utilized a high-density custom-designed array-based comparative genomic hybridization (aCGH) platform [15, 18] to characterize both macro- or micro-CNVs in 131 ALS-related genes (1969 exons) and to define their load in sALS patients compared to control. CNVs in disease-associated genes are more likely to be biologically relevant for ALS and their characterization may have important clinical value for an accurate diagnosis, prognosis prediction and personalized management of this population [19].

## Materials and methods

### Patient samples

A total of 32 Southern Italian patients (17 males and 15 females), with a diagnosis of sALS according to the EL Escorial criteria [20], and 20 controls of Southern Italian patients affected by neurological disorders without diagnosis of ALS, were used in this study. The study was approved by the Ethics Committees of the University of Palermo (document 04/2019, 29 April 2019) and blood samples were collected after an informed consent was signed. Patients were genetically tested for mutations in *SOD1*,* FUS*, *TARDBP* and *C9ORF72* genes [21]. The clinical and genetic characteristics of sALS patients are reported in Table 1.

**Table 1:** Clinical and genetic characteristics of ALS patients

Sample id	Sex	Age	Age of onset	Survival (month)	ΔFS	Site of onset	S/B	Primary diagnosis	Family hystory of NDG disease (yes/no)	NDG	SOD1	TDP-43	FUS	C9orf72
ALS895	M	60	54	51	0.6	Upper limbs	S	ALS	no	-	N	N	N	N
ALS896	F	61	56	censored		Lower limbs	S	ALS	no	-	N	N	N	N
ALS950	M	73	65	58	0.29	Bulbar region	B	ALS-B	yes	Dementia (mother)	N	N	N	N
ALS951	M	64	58	censored	0.78	Bulbar FTD	B	ALS-FTD	no	-	N	N	N	N
ALS952	F	73	75	censored	0.31	Upper Limbs	S	ALS	no	-	N	N	N	N
ALS957	F	67	63	censored	1.2	Upper Limbs	S	ALS	no	-	N	N	N	N
ALS977	F	71	65	censored	1	Cervical spinal cord	S	ALS -S	no	-	N	N	N	N
ALS978	F	41	36	censored	0.88	Lumbo-sacral region	S	ALS -S	no	-	N	N	N	N
ALS979	M	72	67	28	0.33	Bulbar region	B	ALS -B	no	-	N	N	N	N
ALS1010	F	63	56	60	0.53	Lumbo-sacral region	S	ALS -S	no	-	N	N	N	N
ALS1013	F	82	63	censored	0.15	Lumbo-sacral region	S	ALS -S	no	-	N	N	N	N
ALS1112	M	67	62	censored	0.53	Lumbo-sacral region	S	ALS -S	no	-	N	N	N	N
ALS1113	M	58	55	censored	0.5	Cervical spinal cord	S	ALS -S	no	-	N	N	N	N
ALS1114	M	66	59	57		Lumbo-sacral region	S	ALS -S	no	-	N	N	N	N
ALS1115	M	62	58	38	0.4	Lumbo-sacral region	S	ALS -S	no	-	N	N	N	N
ALS1061	M	79	76	17	0.43	Lumbo-sacral region	S	ALS -S	no	-	N	N	N	N
ALS1062	M	79	74	17	1.33	Lumbo-sacral region	S	SLA-S	no	-	N	N	N	N
ALS1063	M	62	54	censored	0.05	Lumbo-sacral region	S	ALS -S	no	-	N	N	N	N
ALS1064	F	68	60	71	0.15	Lumbo-sacral region	S	ALS	yes	Undiagnosed ALS (paternal uncle)	N	N	N	N
ALS1065	F	94	89	25	0.65	Bulbar region	B	ALS -B	no	-	N	N	N	N
ALS1067	M	55	53	censored	0.5	Cervical spinal cord	S	ALS -S	no	-	N	N	N	N
ALS1068	M	69	21	censored		Cervical spinal cord	S	ALS-S slow evolution	no	-	N	N	N	N
ALS1073	F	68	64	34	1.93	Thoracic region	S	ALS -S	yes	Dementia	N	N	N	N
ALS1075	F	54	50	censored	3	Lumbo-sacral region	S	ALS -S	no	-	N	N	N	N
ALS1078	M	56	53	76	0.2	Cervical spinal cord	S	ALS -S	no	-	N	N	N	N
ALS1117	F	81	61	28	1.5	Lumbo-sacral region	S	ALS -S	no	-	N	N	N	N
ALS1118	F	76	72	12	1.1	Lumbo-sacral region	S	ALS -S	no	-	N	N	N	N
ALS1119	M	82	79	12	2.6	Cervical spinal cord	S	ALS -S	yes	Alzheimer (2 brothers) Dementia (1 sister)	N	N	N	N
ALS1142	M	78	75	27	1.64	Lumbo-sacral region	S	ALS-S	yes	Dementia (mother and many uncles)	N	N	N	N
ALS949	M	93	88	73	0.2	Upper Limbs	S	ALS	no	-	N	N	N	N
ALS953	F	75	72	38	0.58	Lower limbs	S	ALS	no	-	N	N	N	N
ALS1017	M	59	65	31	0.88	Cervical spinal cord	S	ALS-S	yes	Alzheimer (father)	N	N	N	exp

*S* Spinal, *B* Bulbar, *N* normal, *M* Male, *F* Female, exp. The molecular profile was performed by ABI Prism 3130XL genetic analyzer

### Design of custom aCGH

Genomic profiling was performed using a high-density and exon-centric array-based comparative genomic hybridization (aCGH) in an 8 × 60 K array format. This array platform, named *NeuroArray* (version 2.0, Agilent Technologies, Santa Clara, CA), allows to detect single/multi-exon deletions and duplications in genes associated to different neurological disorders, including 131 genes related to ALS (Supplementary materials S1_Table 1) [15, 18]. These latter were categorized, according to ALSoD database (https://alsod.ac.uk), in 5 classes: *Definitive* (variants in these genes have been shown to increase the risk of ALS based on a statistical test), *Clinical modifier* (variants in these genes have been linked to a difference in the clinical phenotype of ALS, often disease duration), *Strong evidence* (variants in these genes have been shown to increase ALS risk in well-conducted recent studies, but require replication or resolution of conflicting evidence), *Moderate evidence* (variants in these genes have been associated with ALS in smaller studies or there may be very contradictory evidence) and *Tenuous* (variants in these genes have been associated with ALS in small old studies and have not stood up to replication).

The array design was performed through the Agilent eArray web portal (Agilent Technologies, Santa Clara, CA), which allows to select the regions of interest and identify the “best-performing” probes from the High-Density (HD) Agilent probe library. Chromosomal coordinates of all RefSeq genes were extrapolated using different open-source databases, such as Biomart (http://www.biomart.org/) and UCSC Genome Browser (http://genome.ucsc.edu), according to the Human Genome Assembly (GRCh37/hg19). Exon coordinates of ALS-related genes were selected and formatted using a homemade R script 2 and then uploaded on SureDesign. All probes with similar characteristics (isothermal probes with a melting temperature of 80° C, probe length of about 60-mers) were selected and filtered using bioinformatics prediction criteria according to probe sensitivity, specificity and responsiveness under appropriate conditions. The array was designed to obtain a coverage of at least 3 probes per exon. Additional probes were added with the SureDesign Genomic Tiling option to cover regions inefficiently represented in the Agilent database. A total of 131 ALS-associated risk genes were analyzed with specific oligonucleotide probes to cover 2030 regions (Supplementary materials S1_Table 1).

### Sample preparation

DNA labelling and hybridization on *NeuroArray* were performed according to the manufacturer’s protocol (Agilent Technologies, Santa Clara, CA). Briefly, aCGH analyses of test DNAs were performed against a pooled reference DNA of the same sex (Euro Reference, Agilent Technologies, Santa Clara, CA), both at the concentration of 500 ng, which were double digested with RsaI and AluI for 2 h at 37 °C. Each digested sample was labelled by random priming with the genomic DNA Enzymatic Labelling Kit (Agilent Technologies, Santa Clara, CA), using Cy5-dUTP for patient DNAs and Cy3-dUTP for control DNAs. Labelled products were purified by using the SureTag DNA Labeling Kit Purification Columns (Agilent Technologies, Santa Clara, CA). After probe denaturation and pre-annealing with Cot-1 DNA, hybridization was performed at 65 °C for 24 h in a rotating oven. After hybridization, the array slides were washed and scanned at 3 μm resolution on a G4900DA SureScan Microarray Scanner System (Agilent Technologies, Santa Clara, CA). Test and reference fluorescence intensities were measured for each spot position, and information on the relative copy number of sequences in the test genome compared to the normal genome were extracted.

The aCGH results were analyzed using Agilent’s Feature Extraction software to assess array spot quality. Raw data were normalized, analyzed and visualized based on the human GRCh37/hg19 assembly using Agilent CytoGenomics v. 5.0 and Genomic Workbench v. 7.0 software (Agilent Technologies, Santa Clara, CA, USA) with the following settings: centralization normalization algorithm with a threshold of 6.0; GC correction with a window size of 2 kb; Diploid Peak Centralization; bin size of 10 for detecting aberrant regions or regions of constant CNVs. Aberrant regions were called using the Aberration Detection Method II (ADM-2) with a score threshold of 6.0. Samples with a derivative standard deviation of log2 ratios (DLRS) > 0.3 were discarded to select analysis with high hybridization quality and copy number alterations were considered as true positive events with a minimum of 3 consecutive probe. CNV calls were based on the log2 ratio of direct signal intensity test/control. As default, values between 0.2 and 1.32 were classified as gain/duplications, values between − 0.2 and − 1 were considered as heterozygous deletion, and values < − 1 were considered as homozygous deletions. To increase quality and remove noise signals, we used a cutoff log ratio > 0.5 for both losses and gains. The load of CNV was measured by calculating the total number of CNVs in sALS patients, and unpaired two tailed t-test with a significance p value < 0.05 was applied to compare the difference in means between sALS and controls. A post hoc power analysis, calculated by using G-Power software, starting from the given means of CNVs load of both sALS patients and controls and relative sample size group, revealed a statistical power (1 – β error probability) = 0.8, which represents the minimum accepted level for valid statistical analysis.

**Fig. 1:**
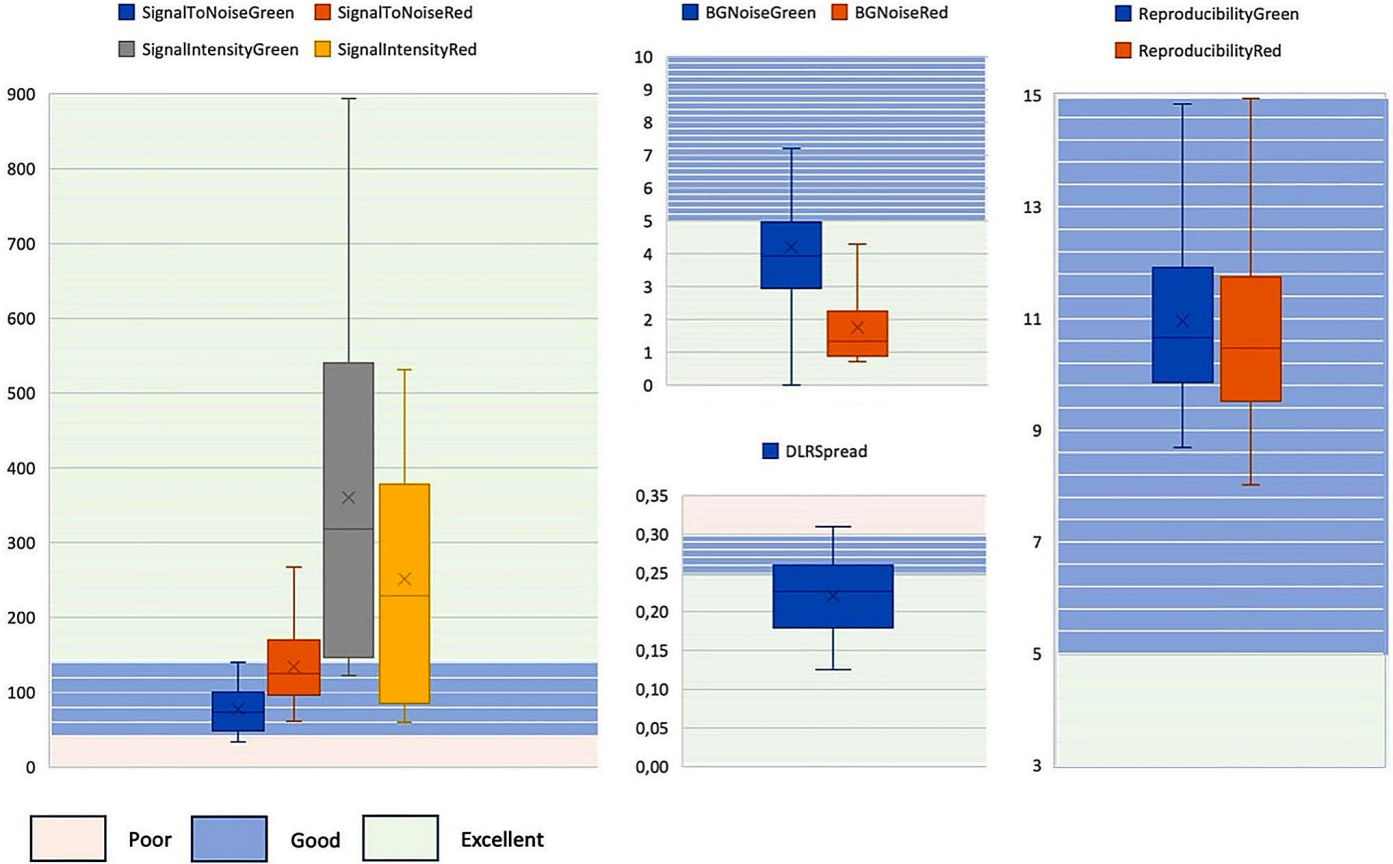
Quality control of aCGH analysis. The following parameters were used to monitor quality of aCGH analysis in each sample: signal-to-noise ratio (SignalToNoise), signal intensity (SignalIntensity), background noise (BGNoise), derivative of log2 ratio spread (DLRSpread), and Reproducibility. Distributions of quality metrics, detected as excellent, good, or poor, are reported as box plots

**Fig. 2:**
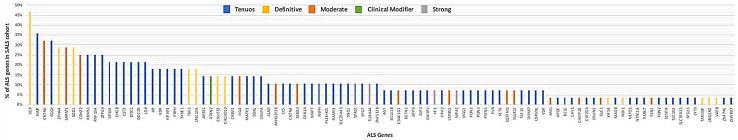
ALS-related genes including CNVs. ALS related genes are highlighted as *Definitive*,* Moderate*,* Strong*,* Tenues* or *Clinical Modifier* according to AlsOD database

**Fig. 3:**
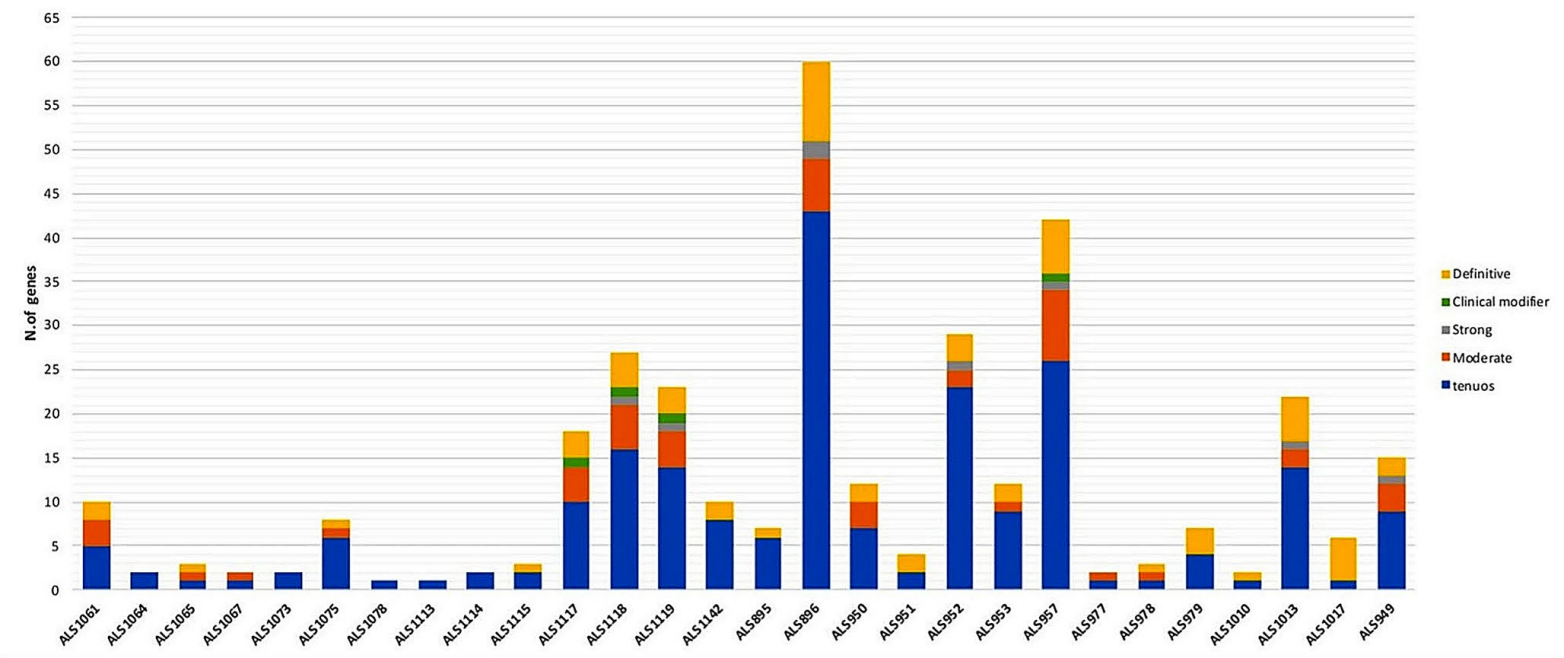
Classification of CNVs in ALS related genes. ALS related genes are categorized as *Definitive*,* Moderate*,* Strong*,* Tenues* or *Clinical Modifier* according to ALSoD database. The graph shows the number of ALS related genes including CNVs in our patient’s cohort

## Results

### Identification of CNVs of ALS-related genes in sporadic ALS patients

To characterize gross- and small-scale CNVs in ALS-related genes, we designed a custom aCGH, named *NeuroArray*, with at least 3 probes/exon, and a median spacing of 0.15 kb in 131 genes previously associated to ALS (Supplementary Materials S1_Table 2). According to ALSoD database [22], the 131 ALS-related genes included 16 *Definitive*, 16 *Moderate*, 4 *Strong*, 94 *Tenuous* and 1 *Clinical Modifier*. We considered values between 0.5 and 1.32 as gain/duplications, values between 0.5 and − 1 as heterozygous deletion, and values < -1 as homozygous deletions. Biologically, a partial loss of the coding sequence may result in a number of different alleles, loss of function or impairment of their regulatory regions; while a complete deletion of the coding sequence could adversely affect gene dosage and protein expression or lead to increased susceptibility to disease. Differently, an increase in gene dosage due to a duplication might lead to overexpression of the gene, and produce expression changes of the relative encoded protein with critical consequences for various cellular processes.

By using *NeuroArray*, we tested 32 southern Italian sALS patients. Array quality values were good/excellent for all parameters considered (Fig. 1).

In 28/32 sALS patients evaluated, we found a total of 643 CNVs (546 gains, 87 losses, 10 deletions) encompassing one or more genes previously associated with ALS (the complete list of CNVs in each patient is shown in Supplementary Materials S2). CNVs concerned 98 out of 131 analyzed ALS related genes (Fig. 2).

Among the CNV-compromised genes, 13 (13.2%) belonged to the class of *Definitive* ALS-genes, 3 (3%) to *Strong*, 13 (13.2%) to the *Moderate*, 68 (69.3%) to *Tenuous* and 1 to *Clinical Modifier* (1%). The number of CNVs found in each patient ranged from 1 to 60, with a median of 7. Although most of CNVs found in each patient encompassed ALS genes classified as *Moderate* (ranging from 1 to 8, median of 1, in 57.1% of patients) or Tenuous (ranging from 1 to 43, median of 4,5, in 100% of patients), several patients comprised CNVs in ALS genes classified as *Strong* (25% of patients) or *Definitive* (ranging from 1 to 9, median of 2, in 75% of patients) (Fig. 3).

**Fig. 4:**
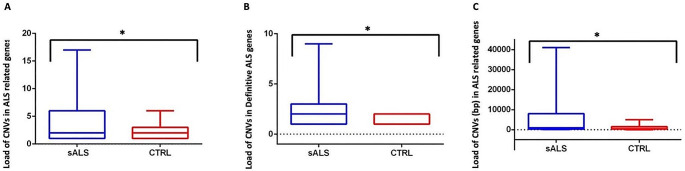
Load of CNVs in sALS patients compared to neurological control samples. Panel A: sALS patients show a higher load of CNVs in ALS-related genes (*Definitive*,* Moderate*,* Strong and Clinical Modifier*) compared to controls (*p*-value = 0,02, unpaired t-test two tailed); Panel B: sALS patients show a higher load of CNVs in *Definitive* ALS genes compared to controls (*p*-value = 0,03, unpaired t-test two tailed); Panel C: sALS patients show a higher total length of CNVs in *Definitive* ALS genes compared to controls (*p*-value = 0,03, unpaired t-test two tailed)

To estimate the collective contribution of CNVs, defined as CNV load, we considered the total number of CNVs and their length in sALS and control patients. Globally, the total number of CNVs events was higher in sALS patients than in control. This increase was significant when considering CNVs in *Definitive*, *Moderate*, *Strong* and *Clinical Modifier* genes (Fig. 4, Panel A; *p* = 0,02), or only *Definitive* genes (Fig. 4, Panel B; *p* = 0,03). The total length of CNVs was 12,091 bp in sALS patients compared to 2,911 bp in control samples. Considering the CNVs in *Definitive*, *Moderate*, *Strong* and *Clinical Modifier* genes, the total length in bp was significantly higher in sALS than controls (Fig. 4, Panel C; *p* = 0,03).

**Fig. 5:**
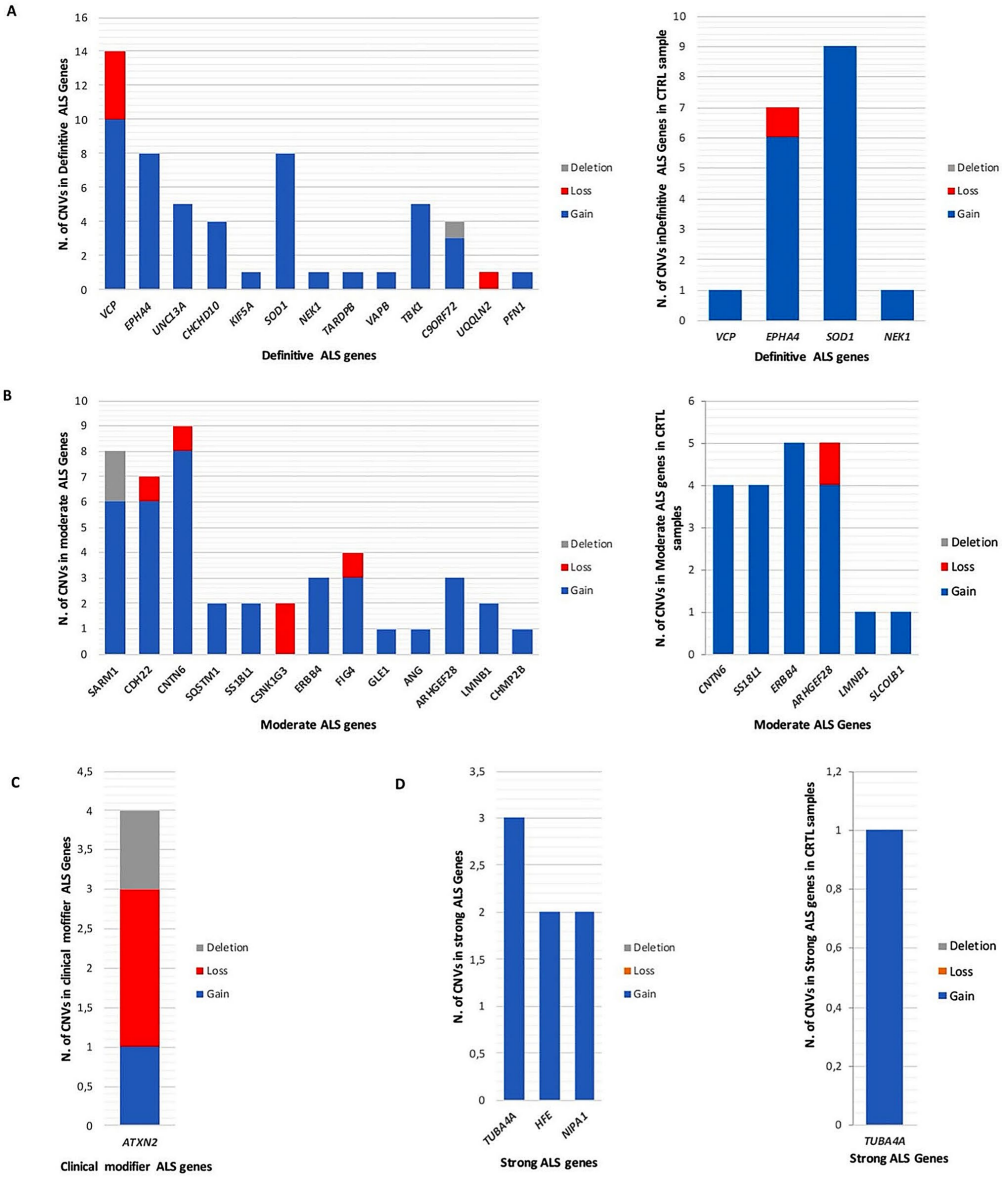
Classification of CNVs based on type and their frequency in sALS patient and control samples; Gain (blue), Loss (red) or Deletion (grey); genes are subdivided based on the classification reported on AlsOD: A = *Definitive*; B = *Moderate*; C = *Clinical Modifier; D= Strong*

Figure 5 shows the number and type of CNVs found in each class of risk-related ALS genes. Interestingly, sALS patients carried 48 gains (ranging from 1 to 10), 5 losses (range: 1–4), and 1 deletion in 13 *Definitive* ALS genes (panel A). A total of 7 gains were identified in 3 *Strong* ALS genes (panel B), 5 losses, 38 gains (ranging from 1 to 8) and 2 deletions in *Moderate* genes (panel C), while 1 gain, 2 losses, and 1 deletion were found in 1 *Clinical Modifier* (panel D). Control samples harbored 17 gains (range: 1–9) and 1 loss in 5 *Definitive* ALS genes, 19 gains (range: 1–5) and 1 loss in 6 *Moderate* ALS genes, and 1 gain in a *Strong* ALS gene.

**Fig. 6:**
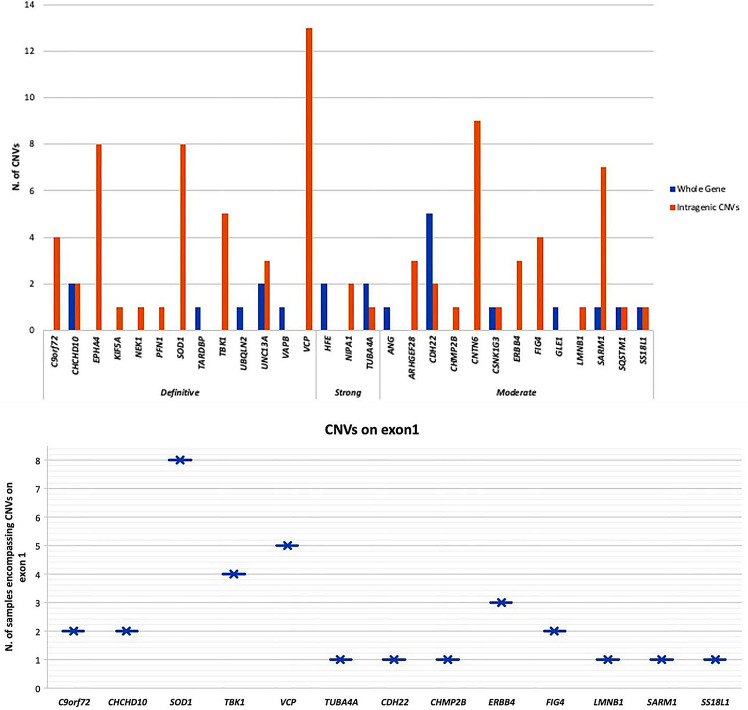
Distribution of large-scale gain and losses, and small-scale (intragenic) copy number aberration in ALS related genes. Gain of exon 1 is the most frequent small-scale (intragenic) CNVs found in 5 *Definitive* ALS genes (*C9orf72*,* CHCHD10*,* SOD1*,* TBK1*,* VCP*), 7 *Moderate* ALS genes and 1 *Strong* ALS gene in 46% of patients

In addition to large-scale amplifications and losses, we observed frequently small-scale (intragenic) copy number aberrations in the coding regions of 5 *Definitive*, 2 *Strong* and 7 *Moderate* ALS genes (Fig. 6). An example is the gain of exon 1 in 5 *Definitive* (*C9orf72*,* CHCHD10*,* SOD1*,* TBK1*,* VCP*), 7 *Moderate* and 1 *Strong* ALS gene detected in 46% of patients (Fig. 6). In control samples, no aberrations encompassing the coding regions of ALS-related genes were observed, whereas intragenic aberrations in *SOD1* and *EPHA4* were equally frequent in sALS and control samples.

Given that previous CNV studies reported population-specific CNVs profiles [23], we searched in sALS patients for the presence of common CNVs in ALS-related genes. CNVs in *VCP* were shared by 46% of the patient cohort, CNVs in *NAIP* by 36%, CNVs in *FGGY* by 32%, and CNVs in *SARM1* were shared by 29% of the patient cohort. Among these, *VCP* and *SARM1* are classified as *Definitive* ALS related gene or Pathogenic according to ALSoD and ClinVar, respectively.

In order to aid research and genetic counselling for the identified CNVs [24], we calculated the penetrance on the basis of the frequency in our population (Supplementary Materials S1_Table 2). In 30% of the patient cohort, we observed a significant amplification for the intervals at Chr1p36.3 (*PLEKHG5*), Chr2q35 (*TUBA4A*), Chr9p13.3 (*VCP*), Chr19p13.1 (*UNC13A*), and ChrXq12 *(AR)* compared to control samples (Table 2).

**Table 2:** CNVs obtained considering an interval penetrance up to 30%

Chr	Start	Stop	% Penetrance	CNV type	Aberration size	Gene
1p36.3	6,545,552	6,556,612	31.25	gain	11,061	PLEKHG5
2q35	220,118,499	220,146,830	31.25	gain	28,332	TUBA4A
2q14.3	64,143,925	64,196,210	40.6	Loss	52,286	VPS54
5p13.2	70,307,077	70,309,855	37.5	gain	2779	NAIP
19p13.1	17,750,714	17,751,468	31.25	gain	755	UNC13A
Xq12	66,941,615	66,941,894	40.6	Gain	280	AR

**Fig. 7:**
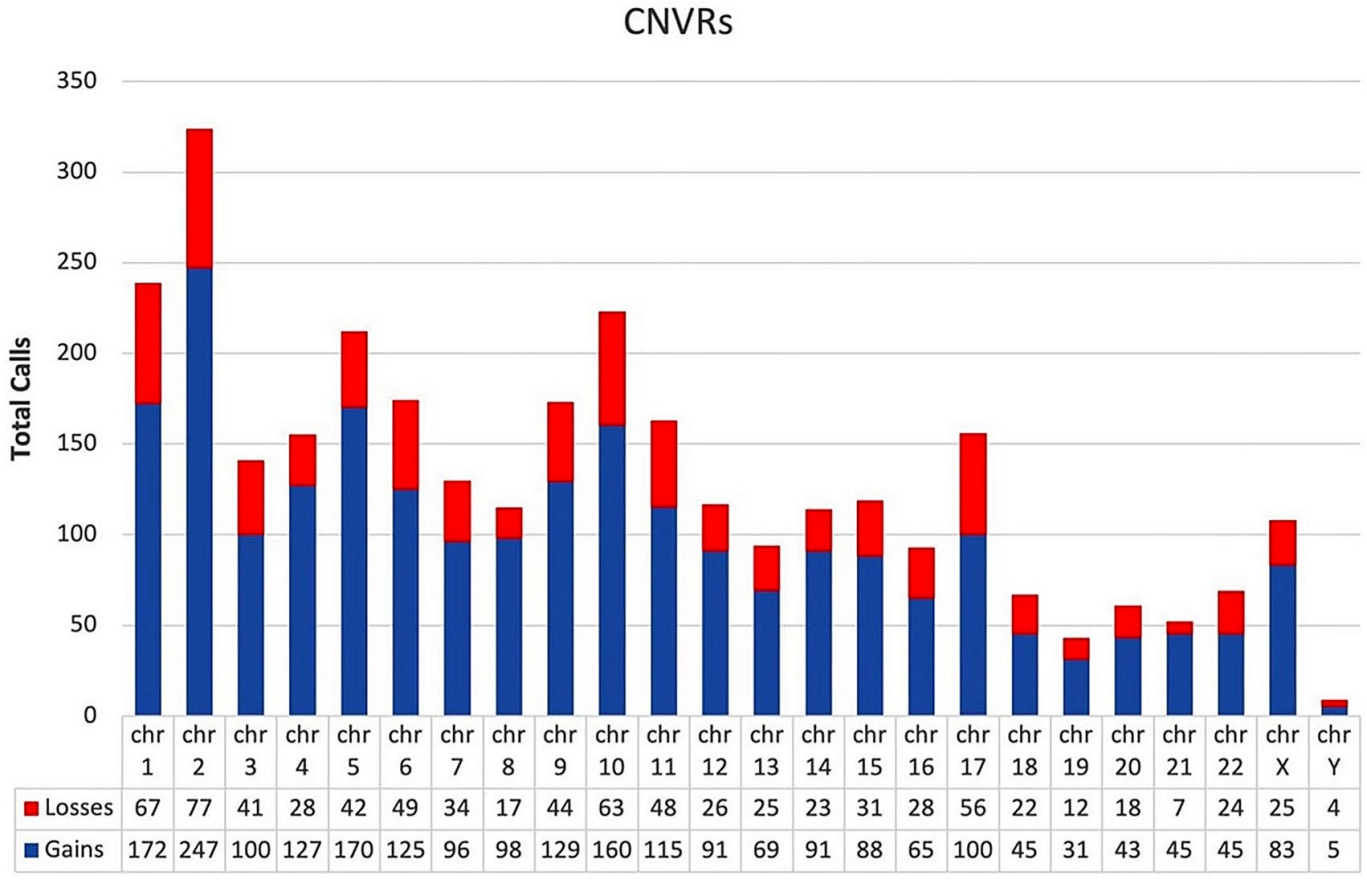
Genomic distribution of CNVRs and their frequency in our cohort. The CNVRs were obtained after merging overlapping CNVs from multiple individuals of our population. CNVR are distributed across all chromosomes. CNVR are listed by CNV type (Loss and Gain)

Additionally, since common CNV regions (CNVRs) are likely to occur at the same genomic locations across different individuals of a homogenous population [25], we investigated the presence of overlapping CNVRs. CNV calls with an intersection of at least 1 kb were grouped into loci that representing all significant CNV calls present in that particular CNVR (Fig. 7). Among 3040 CNVR calls detected (2317 gain and 723 loss) (Supplementary Materials S1_Table 3), 493 CNVRs (353 gain and 140 loss) were common in up to 80% of the patient cohort (Table 3) compared to control samples.

**Table 3:** CNVR common in up to 80% of patients

Chr	Start	Stop	Size	Cytoband	#Probes	#Gains	#Losses	#Calls	#Samples
chr1	146,571,304	231,935,784	85,364,481	q21.1-q42.2	2295	84	32	116	27
chr6	24,658,716	58,613,994	33,955,279	p22.3-p11.2	965	45	28	73	32
chr9	86,322,445	140,893,810	54,571,366	q21.31-q34.3	1828	72	20	92	29
chr16	380,363	35,045,511	34,665,149	p13.3-p11.1	822	32	17	49	26
chr17	25,854,931	80,685,564	54,830,634	q11.2-q25.3	1640	83	40	123	32
chr22	16,197,005	51,106,584	34,909,580	q11.1-q13.3	1120	45	24	69	27

### Correlation between CNVs and patient phenotype

**Fig. 8:**
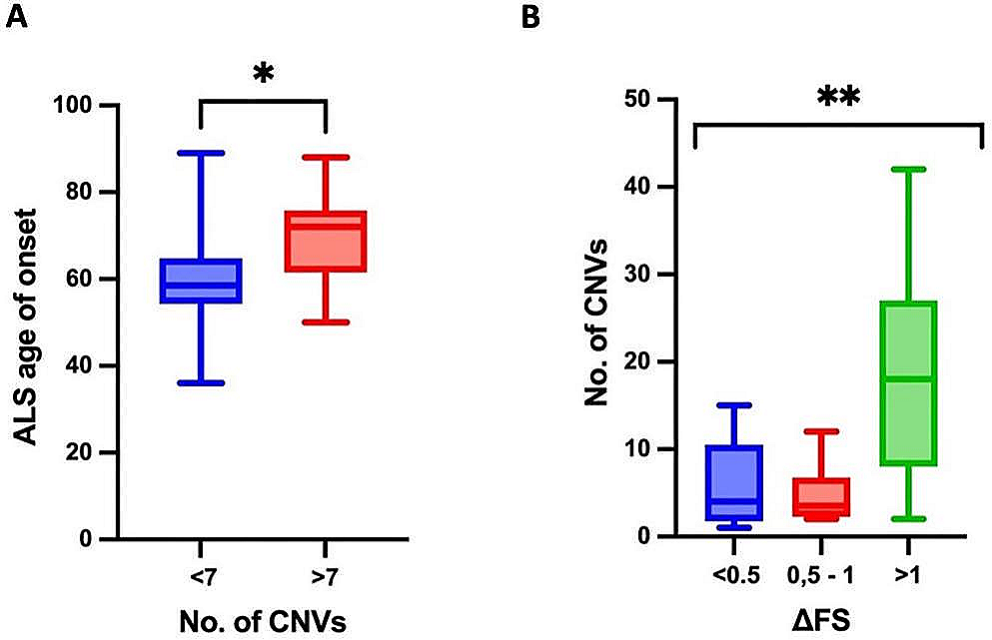
Correlation analysis of identified CNVs with ALS age of onset and disease progression. Panel A: correlation plot between the number of CNVs in ALS genes classified as *Definitive* and age of onset (*p* = 0.02 by unpaired two tailed t-test); Panel B: correlation plot between disease progression score (AFS) and the number of CNVs in ALS genes classified as *Definitive* (*p* = 0,0048 by One-way ANOVA)

To disclose the relationships between identified CNVs and patient phenotype, we correlated the occurrence of CNVs in *Definitive* ALS genes with disease progression score (ΔFS) and patient survival. We observed that patients having a number of CNVs greater than 7 (the median among the patient cohort) had a significant higher (p-value = 0.02) age at onset (Fig. 8, Panel A). Moreover, patients having a slower progression rate (ΔFS < 0.5; average survival time, 51 months) had a similar number of CNVs than patients with intermediate progression rate ( ΔFS score:>0,5 and < 1; average survival time, 41 months), but a lower number of CNVs than patients with a faster progression rate ( ΔFS > 1; average survival time, 21 months) (*p*-value = 0,0048) (Fig. 8, Panel B).

**Fig. 9:**
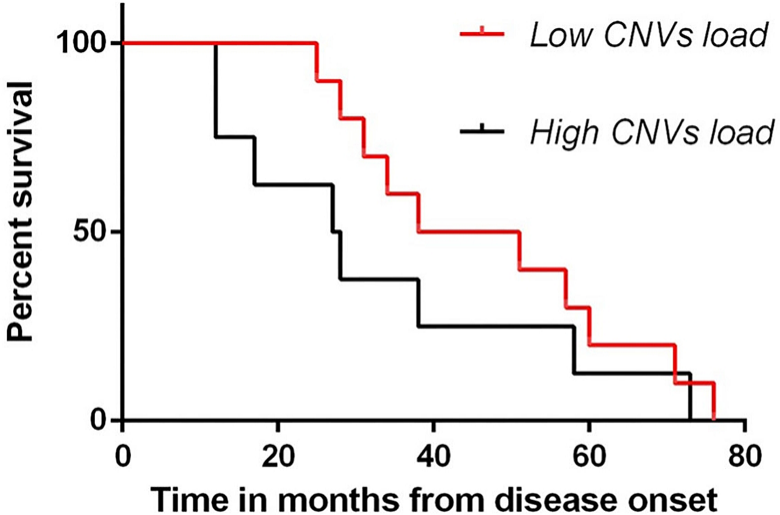
Effects of CNVs load on survival. Kaplan-Meyer analysis showed as patients with low CNVs load had a higher average overall survival (average 47.1), while patients with high CNVs load had a lower average overall survival (average 33.1)

The effects of CNVs load on survival was estimated using a Kaplan-Meyer method. The sALS and control groups were divided according to percentile rank (Low and High CNVs load) (Fig. 9). Although differences between the two groups were not statistically significant, an observable trend showed that patients with low CNV load had a higher average overall survival (average 47.1), while patients with high CNV load had a lower average overall survival (average 33.1).

## Discussion

In this work, we used a high-density, exon-centric aCGH method in sporadic ALS patients to investigate type, frequency, and load of CNVs in the exonic regions of 131 genes previously associated with ALS (18, 25). On the basis of disease risk, these genes are categorized as *Definitive*, *Clinical Modifier*, *Strong*, *Moderate* or *Tenuous* in the ALSoD database [22]. In 87% (28/32) of patients, we observed the presence of a single or multi exons CNVs encompassing ALS related genes. Only few of these CNVs have been previously described, while most are novel. The aberrations stretched over large genomic regions (whole genes) or, more often, produced small-scale intragenic differences. In particular, 59% of patients encompassed aberrations of exon 1 of ALS genes that may affect mRNA stability, pre-mRNA splicing and translation initiation [26, 27]. In 75% of patients (21/28), we observed the presence of CNVs in genes classified as *Definitive* or pathogenic. ALS related genes with the most frequent aberrations included *VCP* (46%), *NAIP* (36%), and *FGGY* (32%). Genomic structural variants in *VCP* are variously associated with ALS risk, younger age of onset and survival [8]. However, in our patient cohort no significant difference in age of onset was observed when those carrying structural variation in *VCP* (mean age of onset 61.9) were compared against those with no structural variation in the *VCP* (mean age of onset 65.4). Similarly, the contribution of CNVs in *VCP* on survival was not significant. Gain of exon 4–5 of *NAIP* was commonly observed in our patient cohort. Classified as a *Tenuous* gene by ALSoD, CNVs on *NAIP* were associated with severe and acute forms of spinal muscular atrophy (SMA) and considered as secondary ‘passenger’ events in ALS pathogenesis [18, 28]. The correlation between CNVs on *NAIP* and age of onset or survival was not significant, although we observed a higher ΔFS in patients with (average ΔFS:1.20) versus those without (average ΔFS: 0.67) *NAIP* aberrations.

Previous publications estimated the penetrance of CNVs specifically for neurological disorders [29, 30]. In our study, patient’s cohort showed significant aberrations with an interval penetrance of up to 30%. The recognition of the incomplete penetrance of CNVs is of extreme importance for genetic counselling, as the same CNV might impact differently in different individuals [29]. However, the availability of large databases of individuals affected and not affected is necessary to estimate the true penetrance rate of these CNVs [31]. Similarly, the CNVRs identified in our cohort and absent in control samples, should be further investigated.

Although the contribution to pathology of individual CNVs is still unknown and will require further studies, our data clearly show an increased CNV load, defined as the total number of CNVs or their length, in sALS vs. control patients. CNV load may also influence disease progression and survival. Indeed, we found that patients with a late onset of disease (average 69 years) have multiple CNVs (> 7) and a faster progression rate (average ΔFS: 1) than patients with a lower number of CNVs (< 7) and early onset of ALS (average 59 years).

Most of the small-scale aberrations found in this study would have not been detected by previous studies utilizing SNPs arrays [12–14]. This methodological approach has limitations in terms of genomic region coverage and resolution. Among the 131 ALS-related genes investigated here, 14,5% were not covered by tagSNPs (14 *Tenuous* genes and 5 *Definitive* genes), 36,6% were covered with less than 5 tagSNPs (40 *Tenuous*, 4 *Moderate*, 1 *Strong* and 3 *Definitive* genes), 23,6% were covered with 5–10 tagSNPs (18 *Tenuous*, 5 *Moderate*, 3 *Strong*, 1 *Clinical modifier*, 4 *Definitive* genes), while only 25% were covered with > 10 tagSNPs (25 *Tenuous*, 6 *Moderate*, 2 *Definitive* genes). Therefore, due to the low coverage of coding regions of ALS-related genes, SNPs array platforms utilized were not fully adequate to investigate the complete repertoire of CNVs in ALS related genes and to assert the presence of only rare CNVs in ALS patients.

## Conclusion

The high number of CNVs identified in ALS-related genes and their significant correlation to disease progression and type or age of onset support the possibility that these structural variants might contribute to the missing heritability in ALS sporadic cases. Although further studies in a larger population of patients with different origins are needed to investigate the individual role of each CNV, our findings have broad implications to understand the polygenic architecture of ALS and may improve the diagnostic, prognostic and therapeutical management of this devastating disease [13, 14].

### Electronic supplementary material

Below is the link to the electronic supplementary material.

Supplementary Material 1

Supplementary Material 2

Supplementary Material 3

## Data Availability

All data generated during this study are included in this published article and the additional files. Raw data from NeuroArray aCGH analysis are available at NCBI’s Gene Expression Omnibus (GEO) at submission number GSE239611.
